# The Ayurvedic drug, *Ksheerabala*, ameliorates quinolinic acid-induced oxidative stress in rat brain

**DOI:** 10.4103/0974-7788.59936

**Published:** 2010

**Authors:** S. S. Swathy, M. Indira

**Affiliations:** *Department of Biochemistry, University of Kerala, Kariavattom, Thiruvananthapuram - 695 581, Kerala, India*

**Keywords:** *Ksheerabala*, Histopathology, lipid peroxidation, oxidative stress, scavenging enzymes, quinolinic acid

## Abstract

One of the mechanisms of neurotoxicity is the induction of oxidative stress. There is hardly any cure for neurotoxicity in modern medicine, whereas many drugs in Ayurveda possess neuroprotective effects; however, there is no scientific validation for these drugs. *Ksheerabala* is an ayurvedic drug which is used to treat central nervous system disorders, arthritis, and insomnia. The aim of our study was to evaluate the effect of *Ksheerabala* on quinolinic acid-induced toxicity in rat brain. The optimal dose of *Ksheerabala* was found from a dose escalation study, wherein it was found that *Ksheerabala* showed maximum protection against quinolinic acid-induced neurotoxicity at a dose of 15 µL/100 g body weight/day, which was selected for further experiments. Four groups of female albino rats were maintained for 21 days as follows: 1. Control group, 2. Quinolinic acid (55 µg/100 g body weight), 3. *Ksheerabala* (15 µL/100 g body weight), 4. *Ksheerabala* (15 µL/100 g body weight) + Quinolinic acid (55 µg/100 g body weight). At the end of the experimental period, levels of lipid peroxidation products, protein carbonyls, and activities of scavenging enzymes were analyzed. The results revealed that quinolinic acid intake caused enhanced lipid and protein peroxidation as evidenced by increased levels of peroxidation products such as malondialdehyde, hydroperoxide, conjugated dienes, and protein carbonyls. On the other hand, the activities of scavenging enzymes such as catalase, superoxide dismutase (SOD), glutathione peroxidase, and glutathione reductase as well as the concentration of glutathione were reduced. On coadminstration of *Ksheerabala* along with quinolinic acid, the levels of all the biochemical parameters were restored to near-normal levels, indicating the protective effect of the drug. These results were reinforced by histopathological studies.

## INTRODUCTION

Sustained adverse interactions between neurotoxins arising from the environment, dietary and lipolytic factors, or from normal metabolism influenced by genetic factors could cause neurotoxicity. One of the common mechanisms for the induction of neurotoxicity is the overproduction of free radicals. Currently, neurorestorative treatment is not available in modern systems of medicine, whereas there are several ayurvedic drugs which have neuroprotective effects. Howerer, there is no scientific validation for these drugs.

**Figure 1:**
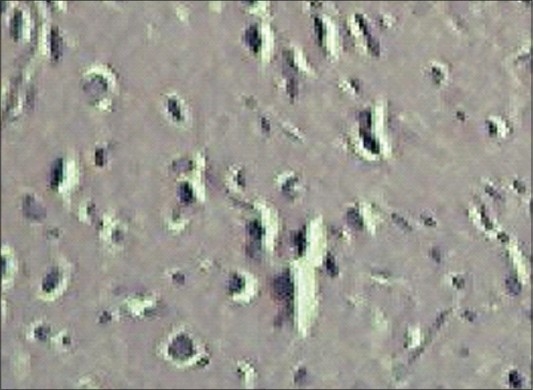
Light microscopic appearance of brain sections obtained using H and E. Microphotograph of brain of the control group; original magnification (× 40). This slide shows the structure of a normal brain; each nerve cell has a distinct nucleus surrounded by cytoplasm.

**Figure 2:**
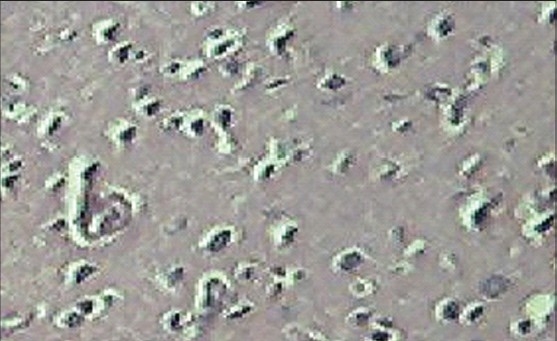
Light microscopic appearance of brain sections obtained using H and E. Microphotograph of brain of the *Ksheerabala* group; original magnification (× 40); the cells were almost similar to that of the control

**Figure 3:**
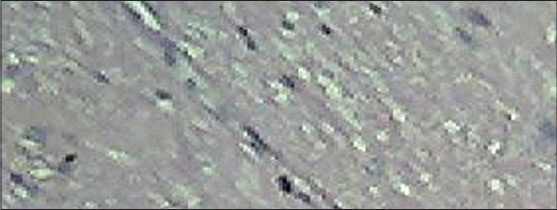
Light microscopic appearance of brain sections obtained using H and E. Microphotograph of brain of the quinolinic acid group; original magnification (× 40). Nerve cells of this slide have undergone degeneration; increased vacuolization can also be observed

**Figure 4:**
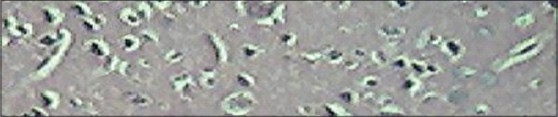
Light microscopic appearance of brain sections obtained using H and E. Microphotograph of brain of the *Ksheerabala* + quinolinic acid group; original magnification (× 40); the cells were almost normal

*Ksheerabala* is an ayurvedic drug used to treat arthritis, central nervous system disorders, and insomnia. The main contents of *Ksheerabala* are Bala (*Sida cordifolia* Linn.), *Ksheera* (cow's milk), and *Thilathaila* (Sesamum oil). The textual reference of *Ksheerabala* is found in *Ashtangahridaya*.[1]

*Sida cordifolia* is a herb from the *Malvaceae* family that is used widely in ayurvedic medicine. Studies conducted by Auddy *et al*.[2] on the antioxidant activity of three Indian medicinal plants used for the management of neurodegenerative diseases showed that *Sida cordifolia* had more potent antioxidant activity than the other herbs. Studies conducted by Dhalwal *et al*.[3] also showed that *Sida cordifolia* is a potential source of natural antioxidants.

There are also reports that milk caseins possess significant antioxidant activity.[4] Studies conducted by Korpela *et al*.[5] have shown that cow's milk had both peroxyl radical-trapping capacity and superoxide radical-trapping capacity.

Sesame oil is derived from the plant species, *Sesamum indicum*[6] and contains several antioxidants[7] including sesamin,[8] tocopherol, sesamolin,[9] and sesaminol. Sesame oil enhances hepatic detoxification of chemicals, reduces the incidence of chemically induced mammary tumors, and protects against oxidative stress.[10] Sesame oil is regarded as a daily supplement to increase cell resistance to lipid peroxidation.[11] Other investigators have also demonstrated the significant neuroprotective activity of sesame oil.[12] Sesame oil modulates oxidative stress and antioxidant status against Fe- induced oxidative damage.[13] Suja *et al*.[14] conducted studies on the antiradical effectiveness of sesame antioxidants, namely, sesamol, lignans, and lignan glycosides isolated from sesame cake extract, and found that all these compounds possessed radical-scavenging activity.

Quinolinic acid is an endogenous neurotoxin that is involved in various neurological disorders. It is used to produce a pharmacological model of Huntington's disease in rats and primates, and has been shown to evoke *N*-methyl-D-aspartate receptor overactivation and oxidative stress.[15] Quinolinic acid has been shown to produce a wide variety of toxic effects in the brain, such as depletion of GABA, excessive increases in cytosolic Ca^2+^ concentration, ATP exhaustion, neuronal oxidative stress, and cell death,[16–18] Quinolinic acid toxicity can also result in caspase-3-like activation and DNA fragmentation.[19]

A review of literature shows that ingredients of *Ksheerabala* have antioxidant properties. One of the mechanisms for the induction of neurotoxicity is through the generation of free radicals. In this study, we have taken quinolinic acid- induced oxidative stress in rat brain as a model system to validate the action of this drug and study its mechanism of action.

## MATERIALS AND METHODS

Female albino rats (Sprague Dawley strain) weighing between 100 and 140 g were divided into four groups of six rats each. Animals were housed in polypropylene cages which were kept in a room that was maintained between 28 and 32°C. The light cycle was composed of 12 h light and 12 h dark. Animals were handled using the Laboratory Animal Welfare Guidelines.[20] Rats were fed with rat feed (Lipton India Ltd.) and food and water were given *ad libitum*. Quinolinic acid dissolved in phosphate-buffered saline (pH.7.4) was administered intraperitoneally, and *Ksheerabala* dissolved in milk was given orally by gastric intubation; control rats received only the vehicle. The duration of the experiment was 21 days and the dose of quinolinic acid was selected on the basis of previous reports.[21] The study protocol was approved by the Institutional Animal Ethics Committee (IAEC -KU-3/2006-2007-BC-MI (15)).

*Ksheerabala* (101) was procured from Kottakkal Arya Vaidyasala, Kottakkal, Kerala, India.

### Standardization data of *Ksheerabala* (101)

- Acid Value: Not more than 70; Iodine value: 30-50; Saponification value: 210-230.
- Loss on Drying: Not more than 1%.
- High Performance Thin Layer Chromatography (HPTLC): Distinct major peaks at Rf: 0.2-0.28, 0.55-065, 0.78-0.85, and around three minor peaks. A unique peak is seen at Rf: 0.45- 0.50. Scanning wavelength: 280 nm, Mobile phase: Benzene Ethyl acetate (6:4), Formic acid: 0.3 mL. Extraction: 20 mL *tailam* heated with 30 mL methanol for 20 min, then kept at –20°C for 12 h. The nonsolidified methanol fraction is filtered, concentrated to 10 mL and used for HPTLC. These data were obtained from the Quality Control Department of Kottakkal Arya Vaidya Sala, Kotttakkal, Kerala.

## EXPERIMENTAL DESIGN

### Experiment I

#### Dose-finding study

Thirty rats were divided into five groups of six rats each:

Group I: Control rats; Group II: Quinolinic acid (55 µg/100 g body weight/day); Group III: *Ksheerabala* (5 µL/100 g body weight/day) + Quinolinic acid (55 µg/100 g body weight/day); Group IV: *Ksheerabala* (15 µL/100 g body weight/day) + Quinolinic acid (55 µg/100 g body weight/day); Group V: *Ksheerabala* (25 µL/100 g body weight/day) + Quinolinic acid(55 µg/100 g body weight/day)

This dose range was selected based on the fact that 8-10 drops of *Ksheerabala* are usually prescribed for an adult patient. After 21 days, the rats were sacrificed and the concentrations of lipid peroxidation products, such as MDA, were determined in the brain.

### Experiment II

#### Detailed study

Group 1: Control rats; Group II: Quinolinic acid (55 µg/100 g body weight/day); Group III: *Ksheerabala* (15 µL/100 g body weight/day); Group IV: *Ksheerabala* (15 µL/100 g body weight/day) + Quinolinic acid (55 µg/100 g body weight/day)

At the end of the experimental period, rats were fasted overnight and sacrificed. The brain tissue was collected for the estimation of various parameters such as the activities of scavenging enzymes, concentrations of lipid peroxidation products and free fatty acids, and the activity of acetyl choline esterase.

### Biochemical analysis

Tissues were extracted according to the procedure of Folch *et al*.[22] and malondialdehyde (MDA) content was estimated by the method of Hiroshi Ohkawa.[23] Hydroperoxide (HP) content was estimated by the method of Mair and Hall[24] and the levels of conjugated dienes (CD) were estimated by the method of Recknagel and Ghoshal.[25] Tissue protein content was estimated by the method of Lowry *et al*.[26] Superoxide dismutase (SOD) activity was assayed by the method of Kakkar *et al*.[27] and catalase activity was assayed by the method of Maehly and Chance.[28] The activity of glutathione reductase (GR) was determined by the method of David and Richard[29] whereas the activity of glutathione peroxidase (GPx) was determined by the method of Lawrence and Burk[30] modified by Agergurd and Jense.[31] Glutathione (GSH) content was determined by the method of Patterzon and Lazarow[32] and free fatty acid content was estimated by the method of Falholt *et al*.[33] Acetylcholine esterase activity was determined by the method of Ellmann and Courtney[34] and the concentration of protein carbonyls was estimated by the method of Abraham and Lester.[35] For the histopathological study, the brain fixed in Bouin's fixative was embedded in paraffin wax and sections were taken in the microtome. Sections were stained by using hematoxylin and eosin after which the pathological changes were examined using a light microscope.

### Statistical analysis

The results were analyzed using a statistical program SPSS/PC+, Version 5.0 (SPSS Inc., Chicago, IL, USA). A one-way ANOVA was employed for comparison among the six groups. Duncan's *post*-*hoc* multiple comparison tests of significant differences among groups were determined, *P* < 0.05 was considered to be significant.

## RESULTS

### Dose-finding study

Quinolinic acid administration increased MDA [Table 1] content in comparison with that of the control group. Administration of *Ksheerabala* along with quinolinic acid was seen to reduce MDA levels with maximum reduction being observed in the group administered 15 µL *Ksheerabala*.

**Table 1:** Effects of various *Ksheerabala* doses on Malondialdehyde content in the brain

Groups	MDA (mmol/100 g tissue)
Control	2.67 ± 0.24
Quinolinic acid	4.73 ± 0.43^a^
5 µL *Ksheerabala* + Quinolinic acid	3.74 ± 0.34^b^
15 µL *Ksheerabala* + Quinolinic acid	2.66 ± 0.24^c^^e^
25 µL *Ksheerabala* + Quinolinic acid	2.74 ± 0.25^d^^f^

Statistical test-one-way ANOVA; Values expressed as mean ± SD; *P < 0.05

a between control and quinolinic acid group

b between quinolinic acid and 5 µL *Ksheerabala* + Quinolinic acid group

c between quinolinic acid and 15 µL *Ksheerabala* + quinolinic acid group

d between quinolinic acid and 25 µL *Ksheerabala* + quinolinic acid group

e between 5 µL *Ksheerabala* + Quinolinic acid group and 15 µL *Ksheerabala* + quinolinic acid group

f between 5 µL *Ksheerabala* + Quinolinic acid group and 25 µL *Ksheerabala* + quinolinic acid group

### Detailed studies

The concentrations of MDA, HP, CD, and protein carbonyls [Table 2] were found to be significantly increased in the brains of the quinolinic acid-treated group compared to the control but there was a significant decrease in the groups treated with quinolinic acid and *Ksheerabala*. There was a decrease in the glutathione content [Table 2] in the quinolinic acid group compared to the Ksheerbala plus quinolinic acid groups.

**Table 2:** Concentrations of malondialdehyde, hydroperoxides, conjugated dienes, glutathione and protein carbonyls in the brain

Groups	Malondialdehyde (mmol/100 g tissue)	Hydroperoxides (mmol/100 g tissue)	Conjugated dienes (mmol/100 g tissue)	Glutathione (mmol/100 g tissue)	Protein carbonyls (nmol/mg protein)
Control	2.18 ± 0.19	34.23 ± 3.12	40.39 ± 3.70	26.71 ± 2.43	1.67 ± 0.16
Quinolinic acid	4.62 ± 0.41^a^	54.78 ± 4.99^a^	68.32 ± 6.23^a^	13.73 ± 1.25^a^	3.35 ± 0.30^a^
*Ksheerabala*	2.42 ± 0.22^b^	33.96 ± 3.08^b^	41.69 ± 3.81^b^	38.33 ± 3.49^b^	1.65 ± 0.15^b^
*Ksheerabala* + Quinolinic acid	3.37 ± 0.29^c^	37.92 ± 3.46^c^	45.90 ± 4.17	17.52 ± 1.59^c^	1.68 ± 0.16^c^

Statistical test-one-way ANOVA; Values expressed as mean ± SD *P < 0.05

a between control and quinolinic acid group

b between quinolinic acid and *Ksheerabala* group

c between quinolinic acid and *Ksheerabala* + quinolinic acid group

The activities of catalase, SOD, glutathione peroxidase, and glutathione reductase [Table 3] were significantly decreased in the brains of the quinolinic acid-treated group when compared to the control. However, on administration of *Ksheerabala*, the activities of all these enzymes were found to increase when compared to the quinolinic acid-treated group.

**Table 3:** Activities of catalase, superoxide dismutase, glutathione reductase, and glutathione peroxidase in the brain

Groups	Catalase#	SOD*	Glutathione reductase@	Glutathione peroxidase@
Control	6.43 ± 0.59	9.17 ± 0.84	52.17 ± 4.75	5.61 ± 0.51
Quinolinic acid	2.09 ± 0.15^a^	2.16 ± 0.19^a^	27.57 ± 2.51^a^	2.13 ± 0.19^a^
*Ksheerabala*	12.23 ±1.12^b^	13.32 ± 1.22^b^	57.75 ± 5.26^b^	7.76 ± 0.32^b^
*Ksheerabala* + Quinolinic acid	3.69 ± 0.35^c^	8.85 ± 0.81^c^	50.67 ± 4.63^c^	4.51 ± 0.41^c^

Statistical test-one-way ANOVA

* *P* < 0.05; Values expressed as mean ± SD

a between control and quinolinic acid group

b between quinolinic acid and *Ksheerabala*group

c between quinolinic acid and *Ksheerabala* + quinolinic acid group

# Units/mg protein [Units: Velocity constant/s] *Units/mg protein [Units: Enzyme concentration required to inhibit the chromogen production by 50% in one min]

@ Units/mg protein [Units: 1 µmol NADPH oxidized/min]

The concentrations of free fatty acids [Table 4] were found to be significantly increased in the quinolinic acid-treated group compared to the control. But the *Ksheerabala* plus quinolinic acid groups showed a significant decrease in the levels of these free fatty acids.

**Table 4:** Concentration of free fatty acids and activity of acetylcholine esterase in the brain

Groups	Free Fatty acids (mg/100 g tissue)	Acetylcholine esterase*
Control	700.79 ± 166.32	11.19 ± 1.02
Quinolinic acid	940.14 ± 86.57^a^	4.40 ± 0.40^a^
*Ksheerabala*	722.69 ± 65.95^b^	16.41 ± 1.57^b^
*Ksheerabala* + quinolinic acid	809.61 ± 73.88^c^	12.04 ± 1.15^c^

Statistical test-one-way ANOVA; *P* < 0.05; Values expressed as mean ± SD

a between control and quinolinic acid group

b between quinolinic acid and *Ksheerabala* group

c between quinolinic acid and *Ksheerabala* + quinolinic acid group

* micro moles of acetylcholine degraded/h/mg protein

The activity of the enzyme, acetylcholine esterase, [Table 4] was found to be decreased in the quinolinic acid-treated group but increased in the *Ksheerabala* plus quinolinic acid groups.

Histopathological studies of the brains of the control and *Ksheerabala*-treated groups showed normal neurons. Increased vacuolization and degenerated neurons could be observed in the quinolinic acid group whereas the neurons were almost normal in the brains of the *Ksheerabala* plus quinolinic acid groups.

## DISCUSSION

Scientific studies using *Ksheerabala* have not been documented in experimental animal models. *In vivo* studies in rats help us to elucidate the mechanism of action and validate the drug. Hence, we have used an animal model with quinolinic acid- induced neurotoxicity to validate the drug, *Ksheerabala*, and also to study its mechanism of action. As quinolinic acid is an endogenous neurotoxin, all the parameters were measured in the brain tissue.

Quinolinic acid-induced neurotoxicity is partially mediated by free radical formation and oxidative stress,[36] and results in increased levels of lipid peroxidation products. [37] A dose-finding study indicated 15 µL/100 g body weight *Ksheerabala* as the optimum dose and although 25 µL/100 g body weight *Ksheerabala* produced higher MDA levels, this difference was not statistically significant. Quinolinic acid has been reported to induce oxidative stress[36] and we too observed increased levels of the products of lipid and protein peroxidation such as MDA, HP, CD, and protein carbonyls, as well as decreased activities of scavenging enzymes such as catalase, SOD, glutathione peroxidase, and glutathione reductase in groups administered with quinolinic acid. The levels of free fatty acids were found to be increased in the quinolinic acid group but significantly decreased after the coadminstration of *Ksheerabala* and quinolinic acid. Free fatty acids are the substrates for lipid peroxidation so that the observed increase in lipid peroxidation product levels may actually be due to the increase in the levels of the free fatty acids themselves. These increased levels of free fatty acids may lead to a change in the membrane architecture.

Studies conducted by Rodriguez-Martinez *et al*.[38] and Cruz- Aguado *et al*.[39] showed that quinolinic acid can affect both the GSH: Oxidized glutathione (GSH: GSSG) ratio and glutathione metabolism. In agreement with these reports, we observed decreased glutathione content and decreased activities of glutathione peroxidase and glutathione reductase in the quinolinic acid-treated group, all of which were increased by the co-administration of *Ksheerabala*.

The activity of the membrane-bound enzyme, acetylcholine esterase, showed decreased activity in the quinolinic acid- treated group. This is in agreement with reports by Boegman *et al*.[40] that an acute injection of quinolinic acid could cause a significant reduction of acetylcholine esterase activity. This reduction could be due to changes in the membrane structure and function of membrane-bound enzymes due to the oxidative stress induced by quinolinic acid. The elevation in the activities of membrane-bound enzymes in the Ksheerbala co-administered groups indicates a change in the fluidity of the membrane and in the functioning of brain. This action of *Ksheerabala* is also observed in the histopathological studies. The main ingredients of *Ksheerabala*: *Sida cordifolia*,[2 3] milk,[4 5] and sesamum oil[6–14] have all been reported to possess antioxidant properties. The synergistic action of all the components might thus may have potentiated its neuroprotective effect.

Thus, it can be concluded from both biochemical and histopathological studies that the *Ksheerabala* reduces the oxidative stress induced by quinolinic acid when administered together by affecting the membrane fluidity and architecture of the brain.
